# Non-invasive transcutaneous vagus nerve stimulation during memory retrieval enhances recollection of emotionally salient memories

**DOI:** 10.1038/s41598-026-53772-1

**Published:** 2026-05-23

**Authors:** Manon Giraudier, Carlos Ventura-Bort, Mathias Weymar

**Affiliations:** 1 Department of Biological Psychology and Affective Science, Faculty of Human Sciences, University of Potsdam, 14476 Potsdam, Germany; 2 Faculty of Health Sciences Brandenburg, University of Potsdam, 14476 Potsdam, Germany

**Keywords:** Locus coeruleus, Memory retrieval, Recognition memory, taVNS, tVNS, Vagus nerve, Neuroscience, Psychology, Psychology

## Abstract

Remembering emotionally significant events is critical for the organisms’ behavior and survival. While the mechanisms underlying the formation of such memories are well understood and closely linked to the vagus nerve and the brain’s arousal system, less is known about its contribution to memory retrieval. The current study tested whether non-invasive transcutaneous auricular vagus nerve stimulation (taVNS) applied during a long-term recognition memory task influenced retrieval of unpleasant and neutral scenes that had been incidentally encoded one week earlier. Results showed that taVNS, compared to a sham condition, selectively enhanced the recollection of unpleasant images. Despite a modest effect size (*d* = *0.33*), these findings provide initial human support suggesting a role of the vagus nerve in emotional memory retrieval, thus extending current theoretical models of emotional memory retrieval and opening a new pathway for memory modulation and non-invasive therapeutic interventions.

**Supplementary Information:**

The online version contains supplementary material available at 10.1038/s41598-026-53772-1.

## Introduction

Remembering emotionally significant events is essential in various aspects of everyday life, as emotional memories guide decision-making, shape social interactions, and influence mental well-being^1,2^. While the mechanisms underlying the formation of such powerful and enduring memories are well understood, less is known about how these memories are retrieved. Understanding these processes, however, is important not only for basic research but also for clinical applications, for example, in post-traumatic stress disorder (PTSD) and neurodegenerative diseases such as Alzheimer’s disease, in which dysregulation of noradrenergic function may lead to memory distortions or deficits^3–6^. In aging populations, a decline in noradrenergic integrity has been associated with reduced emotional memory selectivity, potentially affecting adaptive decision-making^7,8^. Identifying mechanisms that regulate memory retrieval could therefore provide novel strategies to mitigate cognitive deficits and enhance memory function.

One promising target for modulating retrieval processes is the vagus nerve, a central mediator between peripheral physiological states and central arousal mechanisms. Through afferent projections to the nucleus of the solitary tract^9^, the vagus nerve influences the locus coeruleus-noradrenaline (LC-NA) system, which modulates the amygdala and medial temporal lobe (MTL), structures critical for memory formation^10^. Specifically, the amygdala-hippocampus interaction facilitates the encoding and consolidation of emotional memory, while noradrenaline modulates these processes^11,12^. While the LC-NA system is well recognized for its role in encoding and consolidation of emotional memories^13^, its involvement in retrieval is less established. Evidence from animal studies suggests that direct stimulation of the LC during retrieval might enhance memory performance, alleviating forgetting through beta-adrenergic receptor activation, also highlighting the role of noradrenaline in modulating retrieval processes^14^. In humans, functional neuroimaging data indicate that successful retrieval of neutral stimuli encoded in emotional contexts involves LC activity, with functional interactions between the LC and amygdala playing a key role in this process^15^. These findings suggest that the LC-NA arousal system may also be critical during retrieval by influencing attentional and mnemonic processes. Transcutaneous auricular vagus nerve stimulation (taVNS), a non-invasive method that stimulates the auricular branch of the vagus nerve, has emerged as a promising tool for modulating the LC-NA system^16–19^ and its associated memory functions. Recent studies provide evidence that taVNS during encoding enhances recollection-based memory (i.e., the retrieval of specific contextual details associated with the highest level of memory confidence)^20^, particularly for emotionally arousing contents^21,22^, a mnemonic process related to amygdala and hippocampal activity^2,23^. Whether vagal stimulation during retrieval, via LC-NA activation, also improves recollection-based memory for emotional contents, however, is not known so far (but see a recent publication reporting enhanced recall of emotional and neutral stimuli^24^).

The present study aimed to further uncover the mechanisms underlying memory retrieval by investigating whether non-invasive taVNS, applied during a long-term memory recognition task, influenced retrieval of emotional and neutral scenes that had been incidentally encoded one week earlier. Based on prior findings, we expected a memory advantage for emotionally salient stimuli, with this enhancement being driven by recollection-based processes. Since our previous studies have shown that taVNS applied during scene encoding selectively enhanced recollection-based retrieval for unpleasant images, supporting a critical vagal role in the formation of emotional memories, we assumed that, if the vagus nerve-via activation of the LC-NA system-is also involved in memory retrieval (cf.^14^), taVNS would similarly improve recollection-based retrieval, particularly for unpleasant images. In the present work, we observed taVNS-driven increased recollection-based retrieval of unpleasant stimuli. The results suggest that taVNS selectively modulates recollection-based memory processes for emotionally arousing images without any effect on familiarity-based retrieval processes. These results provide novel evidence for a role of the vagus nerve in memory retrieval.

## Results

### Recognition memory performance

The behavioral results for the recognition memory task, as a function of *Category* and *Stimulation*, are summarized in Table 1. As expected, a significant main effect of *Category*, *β* = *0.10, SE* = *0.023, t(73)* = *4.28, p* < *0.001*, indicated significantly higher discrimination index (PR) $[p\left(hit\right)-p\left(false alarm\right)]$ values for unpleasant images compared to neutral images. This effect, however, was not significantly influenced by *Stimulation*, *β* = *0.01,* *SE* = *0.03,* *t(73)* = *0.40,* *p* = *0.69*, indicating that taVNS did not further enhance the memory advantage for unpleasant images. The main effect of *Stimulation*, *β* = *0.02,* *SE* = *0.043,* *t(97.47)* = *0.38,* *p* = *0.71*, was also not significant.

**Table 1:** Mean (standard deviation) of behavioral indices for unpleasant and neutral images encoded under taVNS and sham stimulation. Discrimination index $Pr=p\left(hit\right)-p(false alarm)$*.* Response bias index $Br=\frac{p(false alarm)}{(1-Pr)}$, where Br > 0.5 reflects a liberal response criterion (i.e., a bias toward responding *old*), and lower values indicate a conservative response bias. Recollection index (RI) was calculated separately for unpleasant and neutral images as ${RI}_{Unpleasant}={Pr}_{Recollection\_Unpleasant}{- Pr}_{Familiarity\_Unpleasant}$ and ${RI}_{Neutral}={Pr}_{Recollection\_Neutral}{- Pr}_{Familiarity\_Neutral}$.

	taVNS		Sham	
	Unpleasant	Neutral	Unpleasant	Neutral
Outcome rates				
Hits	0.82 (0.12)	0.64 (0.17)	0.79 (0.12)	0.63 (0.18)
False alarms	0.24 (0.12)	0.17 (0.12)	0.24 (0.16)	0.18 (0.12)
Discrimination index				
Pr	0.58 (0.16)	0.47 (0.19)	0.55 (0.18)	0.45 (0.21)
Response bias index				
Br	0.59 (0.24)	0.33 (0.20)	0.51 (0.22)	0.34 (0.18)
Confidence rates				
Familiarity-based Pr	0.24 (0.14)	0.29 (0.10)	0.26 (0.11)	0.29 (0.12)
Recollection-based Pr	0.58 (0.20)	0.35 (0.20)	0.53 (0.17)	0.35 (0.17)
Recollection index				
RI	0.51 (0.29)	0.22 (0.26)	0.42 (0.22)	0.21 (0.21)

Moreover, for the response bias index (Br), participants demonstrated a more liberal response bias (i.e., higher Br values) for unpleasant images compared to neutral images, *β* = *0.17,* *SE* = *0.03,* *t(73)* = *5.90,* *p* < *0.001*. Although the main effect of *Stimulation* was not significant, *β* =  *− 0.01,* *SE* = *0.05,* *t(104.38)* = − *0.17,* *p* = *0.87,* the interaction between *Stimulation* and *Category* approached significance, *β* = *0.08,* *SE* = *0.04,* *t(73)* = *1.94,* *p* = *0.056*, suggesting a trend toward a more liberal response bias for unpleasant images under taVNS compared to sham stimulation.

### Recognition memory performance based on confidence ratings

Unpleasant images were generally associated with significantly lower Pr values compared to neutral images, *β* = *− 0.05,* *p* = *0.011*. This effect reflects reduced familiarity-based memory performance for unpleasant images specifically under sham stimulation, as this condition served as the reference level in the interaction model. Additionally, a strong main effect of *Memory* was observed, *β* = *0.21, p* < *0.001*, indicating that recollection-based memory was associated with generally better discrimination compared to familiarity-based memory. The main effect of *Stimulation* was not significant, *β* = *0.01, p* = *0.861*.

A significant interaction between *Category* and *Memory*, however, showed that the advantage of recollection over familiarity was greater for unpleasant images than for neutral images, *β* = *0.20, p* < *0.001*, replicating prior findings^23,25,26^. However, no interaction was found between *Category* and *Stimulation*, *β* = *− 0.04, p* = *0.19*, nor between *Memory* and *Stimulation*, *β* = *0.00, p* = *0.996*.

**Fig. 1:**
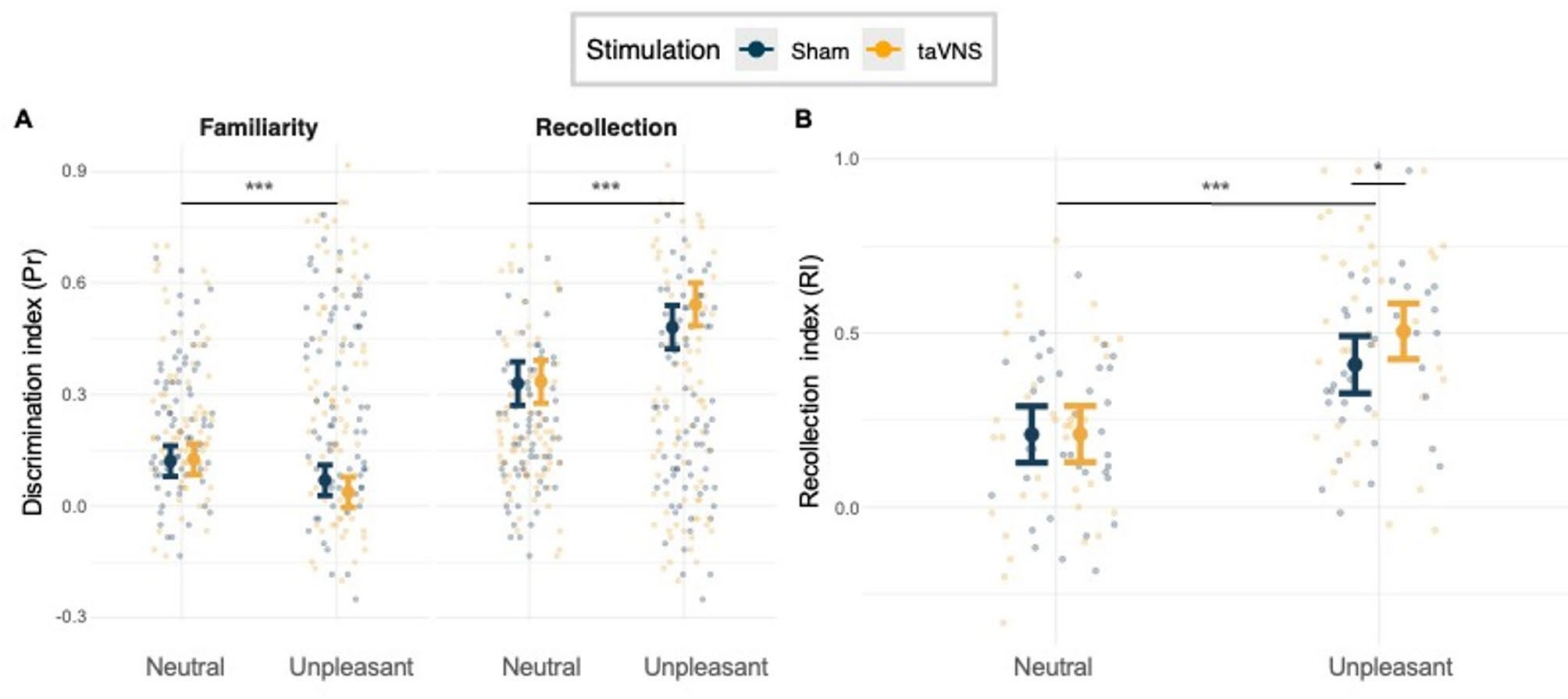
(**A**) The y-axis depicts the discrimination index (Pr), while the x-axis represents the *Category* (unpleasant and neutral images), analyzed for familiarity-based and recollection-based memory under taVNS (orange) and sham stimulation (blue). (**B**) The y-axis shows the recollection index (RI), with the x-axis indicating the *Category* (unpleasant and neutral images), comparing taVNS (orange) and sham stimulation (blue). Bold data points represent model-predicted means (± 95% CI) from the linear mixed-effects model. Faint points show individual participant data.

Critically, a significant three-way interaction between *Stimulation*, *Memory*, and *Category*, *β* = *0.09, p* = *0.019,* suggested that taVNS modulated memory performance specifically for unpleasant images during recollection compared to sham (see Fig. 1A). The simple post-hoc comparison for unpleasant recollection-based discrimination between taVNS and sham was not significant, *β* = *− 0.06,* *p* = *0.14*. The effect size for the difference between stimulation conditions was small, *d* = *0.33*, consistent with a modest effect of taVNS on recollection for unpleasant images^27^. Across both stimulation conditions, recollection-based memory was significantly better than familiarity-based memory for all image categories. The effect was stronger for unpleasant images under taVNS, *Unpleasant*_*Sham*_*: β* =  *− 0.41,* *p* < *0.001, Unpleasant*_*taVNS*_*: β* = *− 0.51,* *p* < *0.001*, aligning with the hypothesis that recollection processes play a greater role in memory for unpleasant images, particularly under taVNS. While the significant three-way interaction indicates that taVNS modulated memory performance as a function of emotional category and memory process, the simple comparison of recollection-based discrimination for unpleasant images between taVNS and sham did not reach significance. This suggests that the effect is not best characterized as an isolated increase in recollection, but rather as a relative shift in the balance between recollection- and familiarity-based memory, which is more directly captured by the Recollection Index (RI) reported below. Moreover, significant variability across participants was observed for the intercept (*SD* = *0.09*) and the slope for *Memory* (*SD* = *0.22*), with a negative correlation between them (*r* = *− 0.75*).

### Recollection index

Because the three-way interaction indicated that taVNS modulated the recollection-familiarity difference as a function of emotional category, we next computed the Recollection Index [RI = ${Pr}_{Recollection}{- Pr}_{Familiarity}]$ to directly quantify this within-condition difference. The RI showed that unpleasant images generally exhibited a greater recollection advantage compared to neutral images, *β* = *0.20,* SE = 0.03, t(71.59) = 6.24, *p* < *0.001*. Although *Stimulation* alone did not significantly influence the recollection advantage, *β* = *0.001, SE* = *0.06,* *t(98.82)* = *0.02, p* = *0.987*, a significant interaction between *Stimulation* and *Category*, *β* = *0.09, SE* = *0.05, t(71.84)* = *2.02, p* = *0.047*, indicated that this advantage for unpleasant images was particularly enhanced under taVNS, suggesting that taVNS selectively modulated recollection-based memory processes for emotionally arousing (unpleasant) images (see Fig. 1B).

### Side effects of stimulation

As expected, participants’ subjective ratings revealed minimal side effects associated with the stimulation (*M* = *1.97, SD* = *1.26*) and there was no indication of significant differences between taVNS and sham stimulation for any of the assessed side effects (*ps* > *0.19*; see Appendix B) (cf.^28^).

## Discussion

In this study, we investigated the role of the vagus nerve in memory retrieval, providing initial human support that vagal activation through non-invasive taVNS may selectively enhance the recollection-based retrieval of emotionally salient memories.

### Enhanced recollection memory for emotional contents under taVNS

As expected, we replicated the well-established memory advantage for emotionally salient stimuli, which were better remembered than neutral ones, particularly through recollection-based memory^12,23,29^, an effect driven by arousal-mediated LC-NA system activation during memory formation^2,13,15^. Critically, such noradrenergic activation also seems to be important for memory retrieval, as taVNS applied during memory reactivation seemed to boost memory (see also^24^ for taVNS effects on free recall, which is generally assumed to rely more strongly on recollection^29^), particularly for emotionally salient stimuli. However, no global effect of taVNS on overall recognition performance was detected, consistent with previous findings showing selective effects on recollection-based memory rather than general recognition sensitivity^20–22^ (likely because overall discrimination performance reflects both recollection and familiarity processes^29^). This pattern aligns with the hypothesis that taVNS modulates LC-NA system activity via afferent projections to the hippocampus-mediated memory system that supports recollection-based memories^12,14,15^. In contrast with familiarity, recollection involves the retrieval of specific contextual details and depends on hippocampal function^2,23^. An important mechanism underlying the vagally-mediated retrieval effects could be the reinstatement of an arousal state during retrieval that mirrors the one experienced during encoding^30^. This is consistent with mediation theory^31^, which proposes that emotional memory advantages stem from cognitive factors such as attention and distinctiveness that shape both encoding and retrieval. Enhanced retrieval might arise when the cognitive and emotional context of encoding is re-engaged, suggesting that taVNS may help reinstate such an arousal context that prioritized emotional stimuli at encoding, thereby selectively facilitating the retrieval of emotionally salient information. From a neurobiological perspective, this reinstatement may be implemented via the LC-NA system. The LC-NA system plays a crucial role in regulating arousal and attention^32,33^, and its activation during retrieval may facilitate access to previously encoded information by re-establishing a neural state similar to that present at encoding^34^.

Moreover, beyond reinstating encoding-related arousal states, taVNS might also enhance memory retrieval by modulating the tonic levels of LC-NA activity. According to adaptive gain theory^32^, the LC-NA system regulates behavior through the interaction of phasic and tonic modes of activity. Moderate tonic LC activity enhances phasic responsiveness to salient or goal-relevant stimuli, thereby improving task engagement and neural gain, whereas high tonic activity leads to nonselective processing and distractibility. Increased tonic activation of the LC-NA system via taVNS could improve the signal-to-noise ratio in neural processing (cf.^32^) and, within this framework, amplify phasic noradrenergic responses to emotionally salient (unpleasant) images while leaving neutral stimuli unaffected. This selective modulation aligns with the GANE model^13,^ which proposes that arousal-related NA release interacts with local glutamatergic signaling to prioritize processing of salient information. Thus, the observed enhancement of recollection for unpleasant images is consistent with LC-NA modulation that optimizes sensitivity to salient emotional cues rather than producing a global facilitation of memory retrieval. Under this framework, the enhanced recollection of unpleasant images under taVNS might be one instance of a broader mechanism wherein LC-NA activity improves task-relevant neural processing. While in this case the goal of the task is to retrieve past experiences, in other contexts, such as attentional control, similar LC-NA modulation may facilitate performance by biasing processing toward relevant stimuli and suppressing irrelevant information^13,35^. In line with this interpretation, the slight reduction in familiarity-based discrimination observed for unpleasant images under taVNS could reflect a functional reallocation of retrieval processes toward more detailed, recollective representations. The recollection effects observed under taVNS may further be modulated by increased neural gain in regions involved in memory retrieval. These include areas of the prefrontal cortex and posterior parietal cortex, which form a network supporting episodic recollection and the reinstatement of stored representation^36^. These regions are known to be innervated by the LC-NA system^32,33^, enabling noradrenergic modulation to enhance cortical excitability and improve the efficiency of retrieval-related neural processing^13^. In addition, through its connection to areas critical for memory formation and emotional processing, such as the amygdala and hippocampus, the LC-NA system may further enhance neural processing within these cognitive and affective domains^10,33^. Although the amygdala is most often linked to emotional memory encoding and consolidation, human neuroimaging studies show that it may also contribute to retrieval and interacts with the LC-NA system during this process (e.g.,^15,23^). Consistent with this framework, animal findings have shown that LC stimulation facilitates memory retrieval via noradrenergic receptor activation^14^, supporting the idea that taVNS may exert its effects through similar pathways. While the current findings are consistent with an LC-NA-mediated modulation of retrieval by taVNS, it should be noted that we did not directly measure activity in the LC (and associated extended network) during retrieval, which needs to be addressed in future studies. Furthermore, from the perspective that taVNS may influence more general cognitive mechanisms, it could also be considered that, for example, stimulation might affect decision thresholds or confidence during recognition, leading participants to adopt a slightly more liberal response criterion for emotionally salient stimuli, as suggested by the trend toward a liberal response bias under taVNS. Moreover, taVNS-induced arousal changes might bias attentional allocation toward emotionally relevant cues, indirectly supporting memory performance without directly altering mnemonic strength.

Overall, while previous studies primarily examined encoding effects of taVNS and reported enhanced emotional recollection memory^21,22^, the current results highlight its role in retrieval. This may suggest that LC-NA modulation has broader functional significance in emotional memory retrieval.

### Future directions, limitations

This study provides support that vagal activation via taVNS enhances emotional memory retrieval, which expands prior work on memory formation (e.g.,^21,22^) and highlights a potential pathway for memory modulation and non-invasive therapeutic interventions^19^. By demonstrating that taVNS selectively improves recollection memory, our findings hold important implications, for instance, for forensic and clinical applications, such as enhancing the accuracy of eyewitness testimony and facilitating memory performance in neurodegenerative conditions such as Alzheimer’s disease. To further extend the current findings, a systematic investigation of stimulation timing-whether applied during encoding, retrieval, or both-would further clarify its differential effects on memory processes. Disentangling the specific contributions of LC-NA activity to encoding versus retrieval and addressing the absence of direct physiological LC-NA markers would provide a foundation for targeted interventions. In this context, future studies may benefit from combining multiple physiological markers, as indices such as salivary alpha-amylase (sAA) have yielded mixed findings in prior taVNS research (e.g.,^20^). This suggests that such effects may be subtle and not consistently detectable in single-study designs. Combining sAA with additional markers previously linked to taVNS and LC-NA activity, such as pupil dilation^18^, may therefore provide a more comprehensive characterization of the underlying neurophysiological mechanisms.

Beyond the LC-NA pathway, taVNS has also been shown to modulate dopaminergic, serotonergic, cholinergic, as well as glutamatergic and GABAergic activity^37^, systems that are involved in memory processes^38^. The interaction between the LC and dorsal raphe nuclei, in particular, may reflect a pathway through which taVNS affects both noradrenergic and serotonergic signaling, jointly contributing to arousal regulation and emotional memory processes. Moreover, dopaminergic signaling may support motivational and goal-directed aspects of retrieval^39^. Future studies combining taVNS with markers of these neurotransmitter systems could help disentangle their contributions to memory retrieval.

Although our statistical models suggest that the observed effects are robust, future studies should minimize potential between-group variability to ensure that the observed effects are attributable to the stimulation itself rather than pre-existing differences between groups. Moreover, as this study employed a single-blind design, potential expectancy or experimenter effects cannot be fully ruled out and future studies should aim to further minimize such influences. Furthermore, to strengthen the interpretability of our findings, future studies should investigate more direct and objective measures of recollection and familiarity (e.g., electrophysiological markers, cf.^21^), as well as alternative indices. Additionally, the observed effect sizes were small-to-medium, indicating a modest effect of taVNS on recollection for unpleasant images. This highlights the importance of replication studies to further validate and refine our understanding of this effect.

## Conclusion

In conclusion, this study demonstrated that taVNS selectively enhanced recollection memory for emotionally salient (unpleasant) stimuli, supporting its hypothesized role in modulating LC-NA system activity. These findings advance our understanding of retrieval processes and underscore taVNS as a promising tool for investigating LC function in memory, with potential applications for non-invasive therapeutic interventions. More broadly, the results contribute to theoretical models of emotional memory by highlighting the role of arousal-related neuromodulation in shaping retrieval processes.

## Methods

### Sample

A total of 80 healthy students from the University of Potsdam participated in the study in exchange for course credits. All participants had normal or corrected-to-normal vision and were German speakers (at least C1 level). Exclusion criteria included neurological or psychiatric disorders, brain surgery, current medication or drug use, pregnancy, a history of migraine and/or epilepsy, cardiac diseases, metal pieces in the body (i.e., a pacemaker), and active implants or physical alterations in the ear (e.g., a cochlear implant). Based on these criteria, five participants were excluded due to reported diagnoses, including anxiety disorders, depression, attention-deficit/hyperactivity disorder and rat phobia. The final sample consisted of 75 participants, who were randomly assigned to either the taVNS group (*N* = *38, 33 female, 5 male, M*_*age*_ = *21.1, SD*_*age*_ = *2.71*) or the sham stimulation group (*N* = *37, 31 female, 6 male, M*_*age*_ = *21.8, SD*_*age*_ = *3.79*). A posteriori power analysis was conducted using G*Power (version 3.1.9.7.), based on the resulting effect size (*d* = *0.33, f* = *0.15, α* = *0.05*) and estimated a power of 0.8 with 80 participants. Each individual provided written informed consent for the study protocol and the application of taVNS in the context of an emotional picture recognition memory paradigm was approved by the Ethics Committee of the University of Potsdam (11/134.24.06.2024, date: 24th June 2024). All data are publicly available (https://osf.io/rqs6p/).

### Experimental procedure

**Fig. 2:**
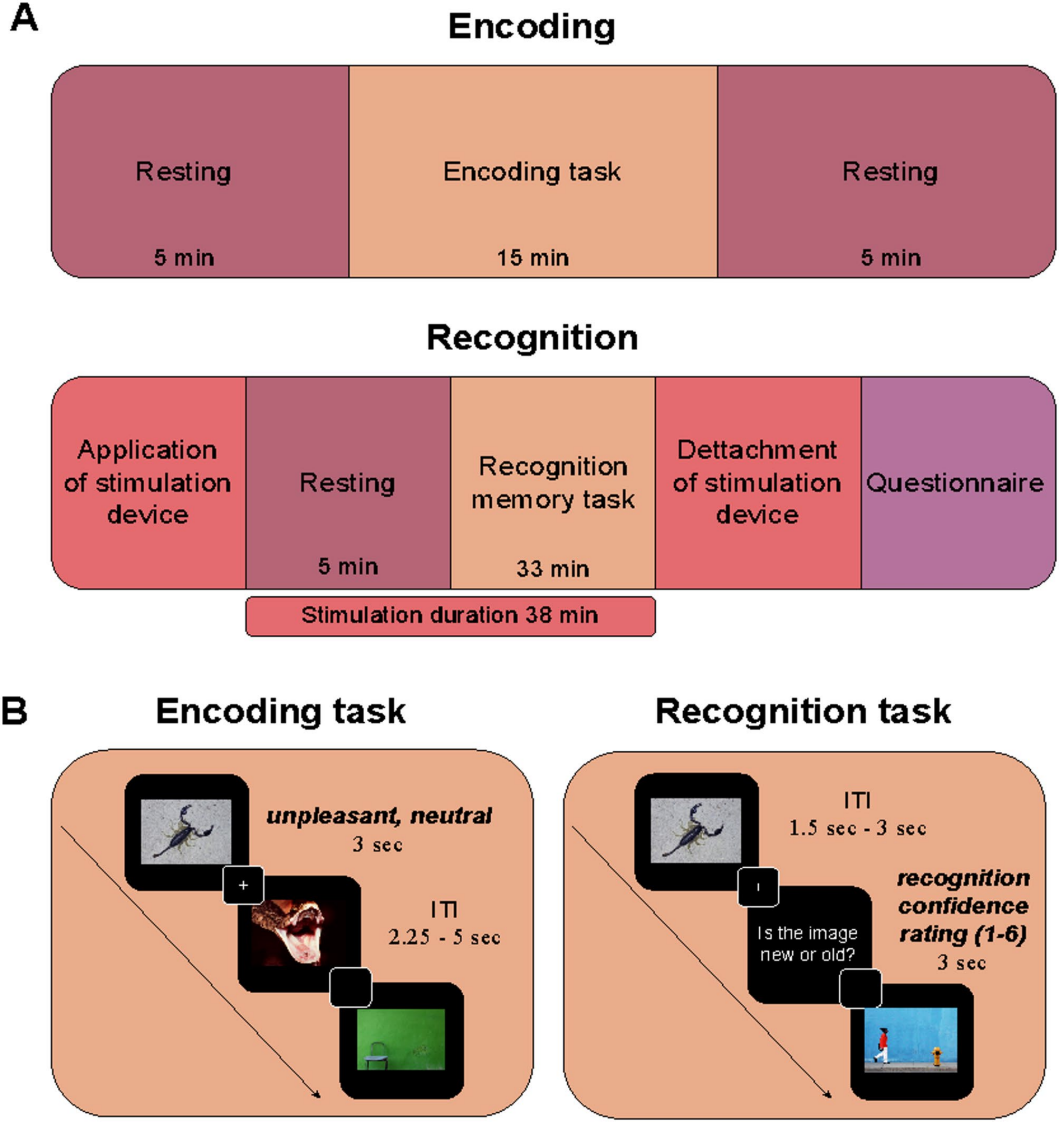
Experimental design consisting of two sessions conducted seven days apart. (**A**) The first session included an encoding task, with 5 min resting phases before and after the task. The second session consisted of a recognition memory task, preceded by a 5 min resting phase with either taVNS or sham stimulation and followed by a side effects questionnaire. (**B**) During encoding, participants attentively viewed neutral and unpleasant images. In the recognition task, participants were presented with previously seen images and new images and rated their memory confidence on a 6-point scale, ranging from 1 (*definitely new*) to 6 (*definitely old*).

A 2-day randomized, single-blinded, between-subject design was employed, with participants randomly assigned to either receive taVNS or sham stimulation. The experiment was conducted over two separate sessions (seven days apart): an encoding session and a recognition session (see Fig. 2A). Stimulation was administered only during the recognition session.

During the encoding session, participants were seated in front of a computer screen and presented with a total of 120 images, consisting of 60 neutral and 60 unpleasant images. Each image was displayed for 3000 ms, with an inter-trial interval between 2500 and 5000 ms. Picture presentation was pseudorandomized with no more than two images from the same category presented consecutively. Before and after the task, participants underwent 5 min resting phases without any image presentation. During the task, participants were instructed to view the images attentively, without being informed about the subsequent memory test, ensuring incidental encoding. One week after the encoding session, participants returned to the laboratory for the recognition session. Upon arrival, the stimulation electrodes were attached to the left ear of participants, and the stimulation intensity was calibrated according to individual sensitivity (for detailed protocol see **Transcutaneous Auricular Vagus Nerve Stimulation** section). Following calibration, participants received 5 min of stimulation without any task. Subsequently, participants performed a recognition memory task, which lasted 33 min and included all of the 120 previously encoded images randomly intermixed with 120 new images, resulting in a total of 240 trials (see Fig. 2B). Each image was displayed on the screen for 3000 ms, preceded by a fixation cross. After each image, participants were asked to assess their recognition memory allowing to differentiate between familiarity and recollection using a 6-point confidence scale, with 1 indicating the picture is *definitely new*, 2 indicating the picture is *probably new*, 3 indicating the picture is *perhaps new*, 4 indicating the picture is *perhaps old*, 5 indicating the picture is *probably old* and 6 indicating the picture is *definitely old*.

After the completion of the task, the stimulation electrodes were removed, and participants completed a side-effects questionnaire (adapted from^43^), for which they had to indicate on a 7-point Likert scale (1 being *not at all* and 7 being *very much*), how much they had experienced headaches, nausea, dizziness, neck pain, muscle contractions in the neck or face, stinging sensations under the electrode, skin irritation at the ear, concentration fluctuations, mood changes, and unpleasant feelings.

### Stimulus material

A total of 240 images selected from the *International Affective Picture System* (IAPS)^44^, the *Nencki Affective Picture System* (NAPS)^45^, and the *EmoMadrid Set*^46^ were used in the experiment. The images were preselected based on their standardized valence and arousal ratings, and included 120 neutral contents (e.g., buildings, neutral landscapes, human faces with neutral expressions; *M*_*valence*_ = *5.23, SD*_*valence*_ = *0.35; M*_*arousal*_ = *4.40, SD*_*arousal*_ = *0.84*) and 120 unpleasant contents (e.g., mutilations, attacks, accidents; *M*_*valence*_ = *2.30, SD*_*valence*_ = *0.71; M*_*arousal*_ = *7.29, SD*_*arousal*_ = *0.98*). Images were counterbalanced across participants by creating eight different image lists. Each list was divided into four sets, with each set containing 30 neutral and 30 unpleasant images. These sets were matched in terms of valence, arousal, and content. During the retrieval task, each set was used either as a previously seen or a novel image, and the assignment of sets to experimental conditions was counterbalanced across participants. Each participant was randomly assigned to one of the eight lists, preventing order or list effects.

### Transcutaneous auricular vagus nerve stimulation

The stimulation device consisted of two titanium electrodes mounted on a holder resembling in-ear headphones, connected to a battery-operated stimulation unit (CMO2, Cerbomed, Erlangen, Germany). In the taVNS condition, the electrodes were positioned on the left cymba conchae, an area innervated exclusively by the auricular branch of the vagus nerve (ABVN)^47^. As in previous studies using taVNS (e.g.,^20,21^), for sham condition, the electrodes were placed on the center of the left earlobe, an area known to be free of vagal innervation^47^. Electrical stimulation was applied in alternating 30-s cycles of stimulation and rest. The device delivered stimulation with a pulse width of 250 µs and a frequency of 25 Hz. Stimulation intensity was individually calibrated to be above the sensory threshold but below the pain threshold (cf.^20,21^). The stimulation intensity differed between the taVNS (*M* = *1.12, SD* = *0.615*) and the sham conditions (*M* = *1.49, SD* = *0.481*), *t(68.10)* = *2.93, p* = *0.005*. However, this difference was not expected to influence the results, as stimulation intensity has generally shown no significant effects on physiological or behavioral outcomes (e.g.,^48–50^). As expected, the inclusion of stimulation intensity as a covariate in the statistical models also did not change the reported memory effects (see Appendix A).

### Statistics

All statistical analyses were carried out in the R environment (version 4.3.0.).

#### Self-report measures

To test whether the reported side effects differed between taVNS and sham stimulation, t-tests were performed for each reported subjective symptom, separately.

#### Recognition memory performance

To assess the effects of taVNS on recognition memory performance, the discrimination index Pr was defined as $Pr=p\left(hit\right)-p(false alarm)$*,* where higher Pr values indicate better discrimination between old and new images. The response bias index Br was calculated as $Br=\frac{p(false alarm)}{(1-Pr)}$, where Br > 0.5 reflects a liberal response criterion (i.e., a bias toward responding *old*), and lower values indicate a conservative response bias^51^. Responses were categorized as *old* if rated 4–6 on the confidence scale and *new* if rated 1–3. The Pr and the Br index were analyzed using a linear mixed-effects model, with fixed effects of *Stimulation* (taVNS vs. sham) and *Category* (unpleasant vs. neutral), including their interaction and a random intercept for participants.

#### Recognition memory performance based on confidence ratings

Memory performance was divided according to responses on the 6-point confidence scale to differentiate between recollection- and familiarity-based retrieval processes. This classification followed the dual-process signal detection framework, which posits that recollection is a threshold-like process typically associated with the highest-confidence recognition judgments, whereas familiarity is a graded signal reflected by intermediate confidence levels^29,40–42^. Consistent with this literature and prior taVNS research^21^, responses with a rating of 6 (*definitely old*) were categorized as recollection-based, while responses with ratings of 4 (*perhaps old*) and 5 (*probably old*) were categorized as familiarity-based memory. The Pr index was analyzed using a linear mixed-effects model, with fixed effects of *Stimulation* (taVNS vs. sham), *Category* (unpleasant vs. neutral), and *Memory* (familiarity vs. recollection), including their interactions. Effect sizes for post-hoc comparisons are reported as Cohen’s d. Participant intercepts and slopes for *Memory* were modeled as random effects to account for individual variability in response patterns. The selected random-effect structure included theoretically relevant variance components and was supported by the data. Data points falling outside 1.5 times the interquartile ranges above the third quartile or below the first quartile (1% of the data) were excluded from further analysis^52^.

#### Recollection index

To clearly visualize the findings from the linear mixed-effects analysis of recognition memory performance based on confidence ratings, particularly regarding the interplay between *Stimulation*, *Memory*, and *Category*, a Recollection Index (RI) was calculated. This index represented the relative advantage of recollection-based memory over familiarity-based memory, defined as ${RI}_{Unpleasant}={Pr}_{Recollection\_Unpleasant}{- Pr}_{Familiarity\_Unpleasant}$ and ${RI}_{Neutral}={Pr}_{Recollection\_Neutral}{- Pr}_{Familiarity\_Neutral}$. By computing this difference, we isolated the extent to which recollection processes contributed to discrimination performance relative to familiarity-based memory, providing a measure of the recollection advantage for both neutral and unpleasant images. The RI was analyzed using a linear mixed-effects model, with fixed effects of *Stimulation* (taVNS vs. sham) and *Category* (unpleasant vs. neutral), including their interaction and a random intercept for participants.

#### Receiver operating characteristic (ROC) analyses

To further investigate the differential effects of taVNS on familiarity and recollection processes, individual behavioral responses were used to create ROC curves using the ROC toolbox^53^ to extract indices of recollection and familiarity. These indices were analyzed using a linear mixed-effects model, with fixed effects of *Stimulation* (taVNS vs. sham), *Category* (unpleasant vs. neutral), and *Memory* (familiarity vs. recollection), including their interactions. Results of this analysis, however, did not reveal additional information and were therefore not included in this paper (results can be found on the Open Science Framework https://osf.io/rqs6p/).

## Supplementary Information

Supplementary Information.

## Data Availability

All data and analysis scripts are publicly available (https://osf.io/rqs6p/).
